# Supplementary material for: OMAmer: tree-driven and alignment-free protein assignment to subfamilies outperforms closest sequence approaches
Source: Bioinformatics. 2021 Mar 31;37(18):2866–73. doi: 10.1093/bioinformatics/btab219 (PMC8479680; doi:10.1093/bioinformatics/btab219)
Supplement: btab219_supplementary_data [file btab219_supplementary_data.pdf]

Supplementary material for OMAmer

Supplementary methods

k-mer integer encoding

The integer encoding of a *k*-mer x formed of k numerical characters x_i, ordered from $i = 1$ to $i = k$, from an alphabet A (A:0, C:1, …, Y:20) is defined as:

$$\sum_{i=1}^{k} x_i |A|^{k-i}$$

OMAmer-score computation

The coarse alignment-free similarity (1) is measured as the number of intersecting *k*-mers between the query *k*-mers Q and the HOG specific *k*-mers H. H includes *k*-mers specific to the HOG descendants but excludes the ones conserved in its ancestors. To compute (1), the number of intersecting *k*-mers between the query *k*-mers and each HOG ancestral *k*-mer set (the *k*-mers inferred to have arisen in the HOG) is retrieved from the precomputed *k*-mer table. Then, these counts are cumulated from leaves to root by adding the highest child HOG *k*-mer count to the current HOG count at each multifurcation.

$$|Q \cap H| \quad (1)$$

To account for the different sizes (number of different *k*-mers) of reference HOGs and the query composition bias, the expected number of shared *k*-mers between the query and the HOG observed in absence of homology, *i.e.* by chance, (2) is subtracted from (1) (3). OMAmer proposes a parametric (default OMAmer-score) and a non-parametric approach (sensitive OMAmer-score) to compute (2).

$$E(|Q \cap H|) \quad (2)$$
$$|Q \cap H| - E(|Q \cap H|) \quad (3)$$

In the parametric approach, (2) is calculated as the number of query *k*-mers $|Q|$ multiplied by the probability to observe one query *k*-mer x_q in H.

$$E(|Q \cap H|) = |Q| \, P(x_q \in H)$$

This probability is the inverse probability of not observing one x_q in H.

$$P(x_q \in H) = 1 - (1 - P(x_q))^{|H|}$$

The probability of observing $x_q$in a HOG of size one, *i.e.* with one *k*-mer, $P(x_q)$ is approximated as the mean frequency of query *k*-mers inside the *k*-mer table (the average fraction of HOGs containing each query *k*-mer x_i [remember that each *k*-mer can only be stored once per root-HOG]).

$$P(x_q) = \frac{1}{|Q|} \sum_{i=0}^{|Q|} freq(x_i)$$

In the non-parametric approach, (2) is simply (1) obtained from a random permutation of the query sequence. In an attempt to conserve some local composition bias, the permutation is performed by shuffling windows of size six in addition to shuffling individual amino acids within each such window. Note that this approach additionally corrects for HOG composition biases.

Finally, to make the OMAmer-score comparable across queries, (3) is divided by $|Q|$, from which was subtracted the number of query *k*-mers shared with more ancestral HOGs.

$$OMAmer - score = \frac{|Q \cap H| - E(|Q \cap H|)}{|Q| - |Q \cap ancestors(H)|}$$

Datasets and software parameters

OMAmer was compared with two closest sequence methods lying at different extremes of the speed-accuracy tradeoff: DIAMOND (v0.9.14) and Smith-Waterman, respectively. Due to the computational cost of performing Smith-Waterman alignments, we used pre-computed alignments from OMA (January 2020) (Altenhoff *et al.*, 2018). DIAMOND databases were built with default parameters, and searches for the most similar sequence were performed with effectively no significance requirement (E-value set to 1e6). The OMAmer *k*-mer table was built with a *k*-mer size of 6.

OMAmer directly yields family and subfamily predictions. For Smith-Waterman and DIAMOND, each query was assigned to the family and most specific subfamily of its closest reference protein. To obtain multiple precision-recall values, predictions were computed for multiple score thresholds: E-values of 1e-322 to 1e6 for DIAMOND, alignment scores of 1 to 5,000 for Smith-Waterman and OMAmer-scores of 0 to 0.99.

To make family-level assignments comparable and well differentiated from subfamily-assignments, we selected HOGs from OMA (January 2020) defined at the *Metazoa* and *Viridiplantae* taxonomic

levels as root-HOGs (families), and their sub-HOGs as subfamilies. To avoid low-confidence families, we further filtered out root-HOGs with less than six proteins. We picked *Metazoa* because it is one of the largest clades in OMA and *Viridiplantae* due to the high number of duplications and thus subfamilies in this clade. Note, due to the addition of *Branchiostoma lanceolatum* in the January 2020 OMA release, we removed it from the reference database used (before the *k*-mer index precomputation) to keep the same evolutionary distance existing between *Branchiostoma floridae* and reference proteomes of the previous OMA release (June 2019).

Then, we selected six species as experiment targets picked because they stand as outgroups of large clades in OMA and thus display some variability in divergence ages to reference species. Platypus, spotted gar and amphioxus were selected in *Metazoa*, while Gray rockcress, wine grape and *Amborella trichopoda* were chosen in *Viridiplantae* (Supp. Table 2). Clade-specific root-HOGs used to build the negative query set were picked at the *Bacteria* taxonomic level.

The *Metazoa* reference dataset included 1,309,488 proteins from 201 species organized in 235,983 HOGs and including 12,178 root-HOGs. The *Viridiplantae* reference dataset included 554,389 proteins from 63 species organized in 304,838 HOGs and including 8,652 root-HOGs. The query datasets (proteomes) included 5,811, 7,7227,387, 6,239, 7,219 and 5,931 proteins of platypus, spotted gar, amphioxus, gray rockcress, wine grape and *Amborella trichopoda* species, respectively. 4,952, 6,308, 5,803, 5,261, 5,712 and 4,057 queries belonged to a sub-HOG in addition to the root-HOG.

Supplementary figures

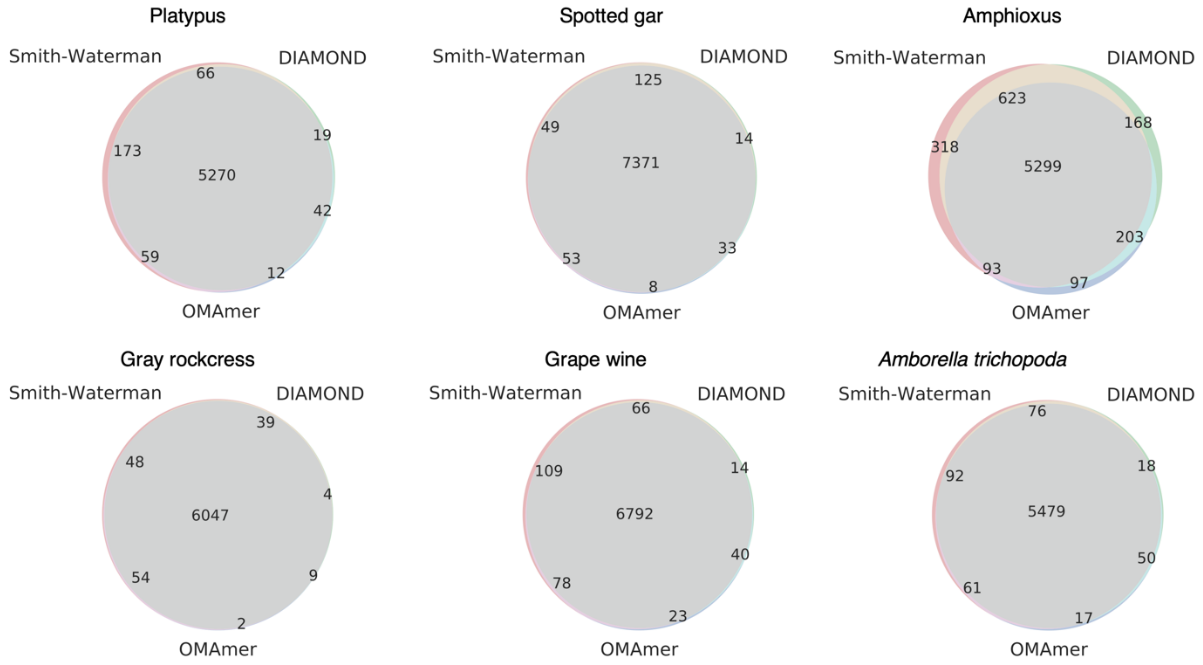

Supp. Fig. 1. Number of family-level TP queries overlapping between methods. TP sets were defined at $F1_{max}$ for DIAMOND and OMAmer and at the minimum score (1) for Smith-Waterman alignments. These queries were used to assess subfamily assignment.

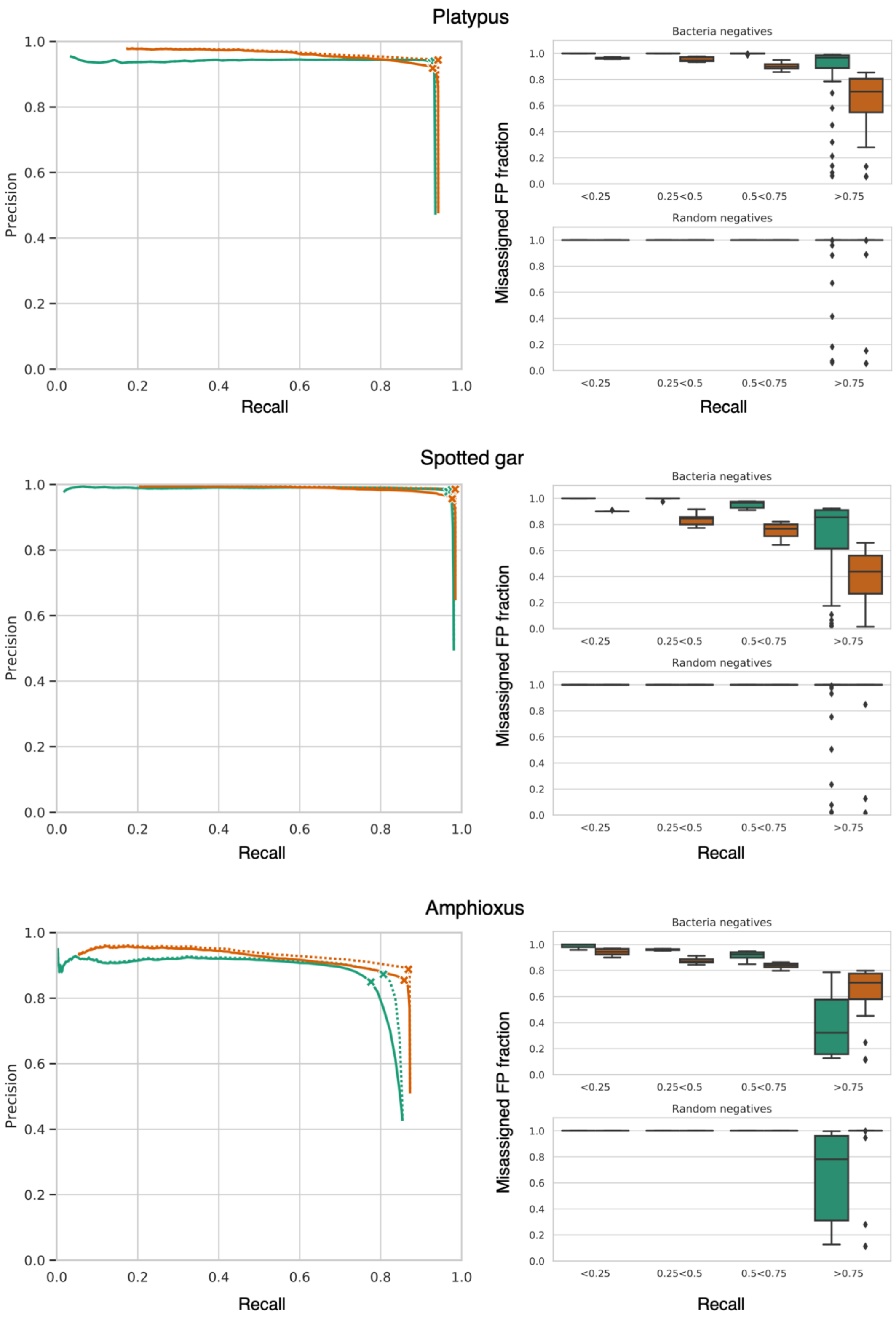

Platypus
Precision
Recall
Bacteria negatives
Random negatives
Misassigned FP fraction
<0.25
0.25<0.5
0.5<0.75
>0.75
Spotted gar
Precision
Recall
Bacteria negatives
Random negatives
Misassigned FP fraction
<0.25
0.25<0.5
0.5<0.75
>0.75
Amphioxus
Precision
Recall
Bacteria negatives
Random negatives
Misassigned FP fraction
<0.25
0.25<0.5
0.5<0.75
>0.75

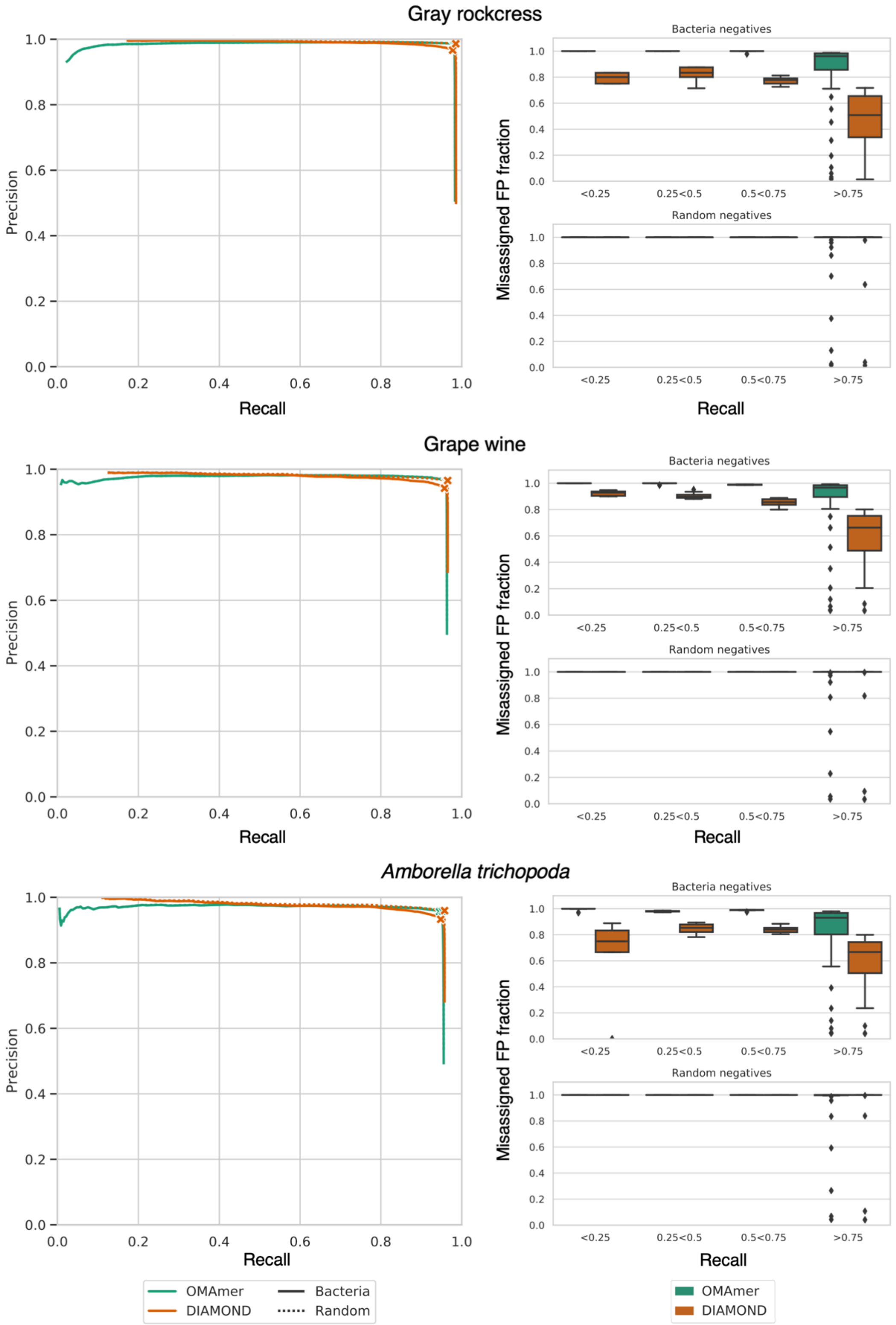

Supp. Fig. 2. Comparison of family assignments between OMAmer and DIAMOND across negative datasets. (Left) Each curve displays the range of trade-offs between precision and recall when varying the threshold on the OMAmer-score or on the DIAMOND E-value. The curves labeled *Bacteria* refer to analyses using bacteria-specific sequences as negatives whereas those labeled *Random* refer to using random sequences as negatives. Crosses indicate the location of F1max values. (Right) Fraction of FPs coming from the misassignment of positive sequences.

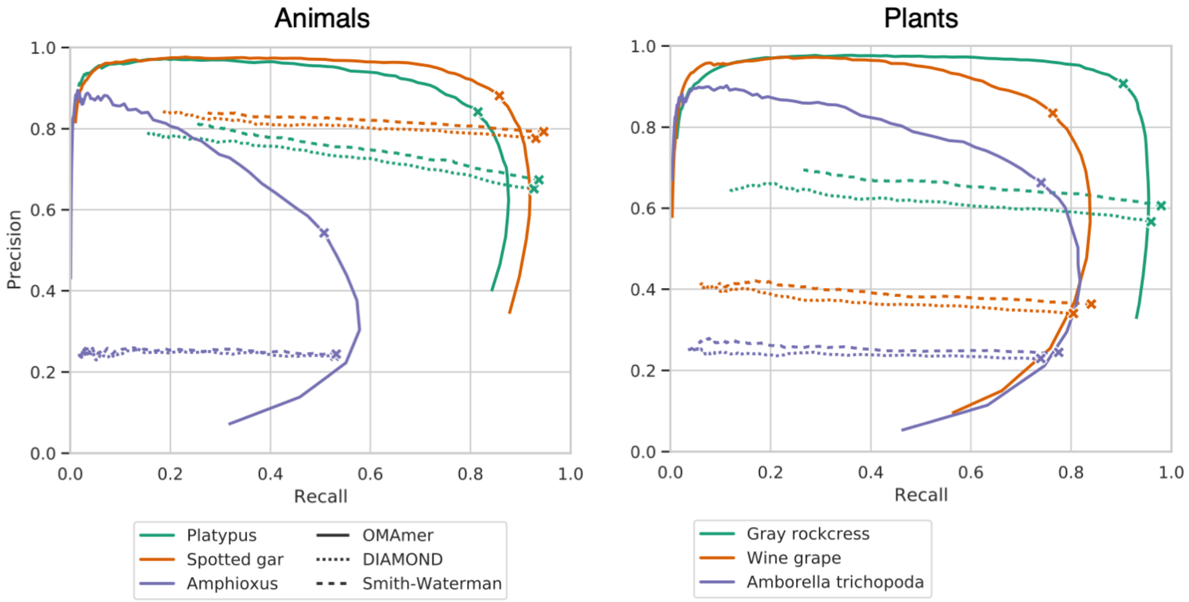

Supp. Fig. 3. Comparison of subfamily assignments with OMAmer and by closest sequence (Smith-Waterman and DIAMOND). Each curve displays the range of trade-offs between precision and recall when varying the threshold either on the OMAmer-score, on the DIAMOND E-value or on the Smith-Waterman alignment score. These results were computed using the more stringent validation procedure. $F1_{max}$ values are annotated with crosses.

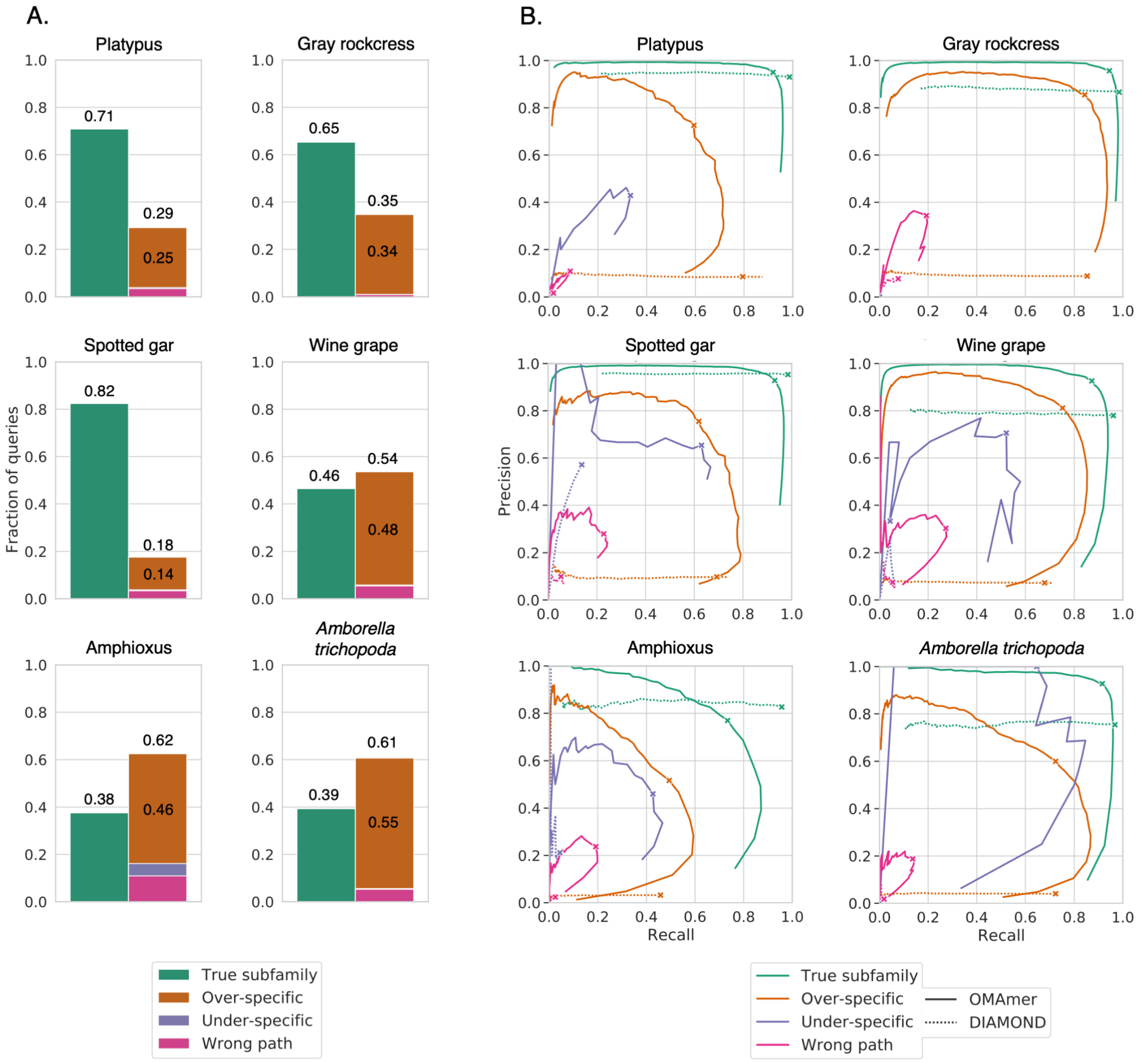

Fig. 4. Frequency of closest sequence configurations defined in Fig. 1 and OMAmer accuracy for each. A. The closest sequence to a query was often found in another subfamily. Smith-Waterman alignments were used as proxies for closest sequences. B. These results were computed using the more stringent validation procedure (*See methods*). Each curve displays the range of trade-offs between precision and recall when varying the threshold on the OMAmer-score and on the DIAMOND E-value. They were computed by breaking down queries by closest sequence configurations as in panel A, before the validation procedure itself. $F1_{max}$ values are annotated with crosses. Crosses indicate the location of $F1_{max}$ values. “Over-specific” $F1_{max}$ values are specifically annotated.

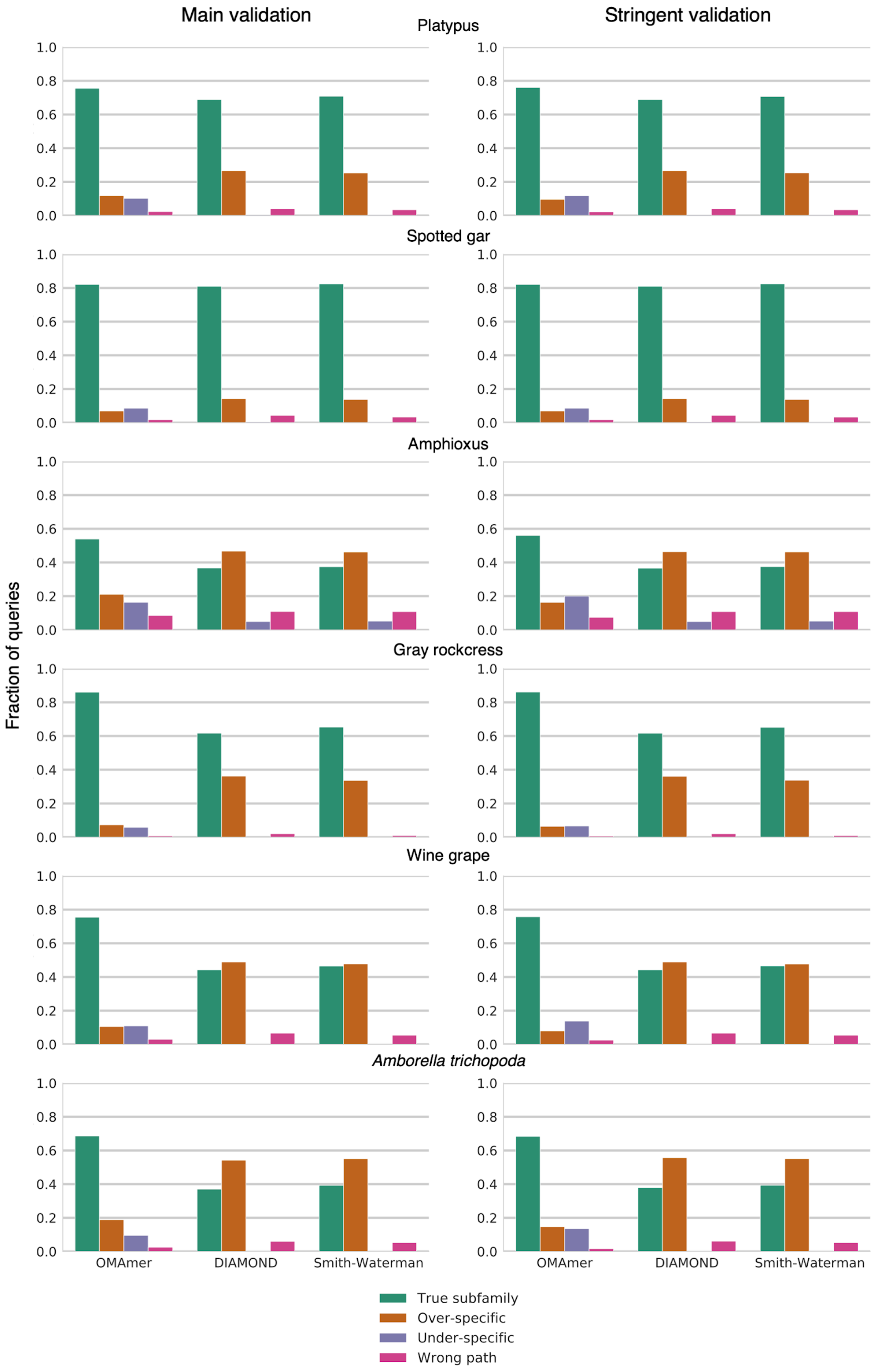

Supp. Figure 5. Partitioning of subfamily assignments at $F1_{max}$ into closest sequence configuration.

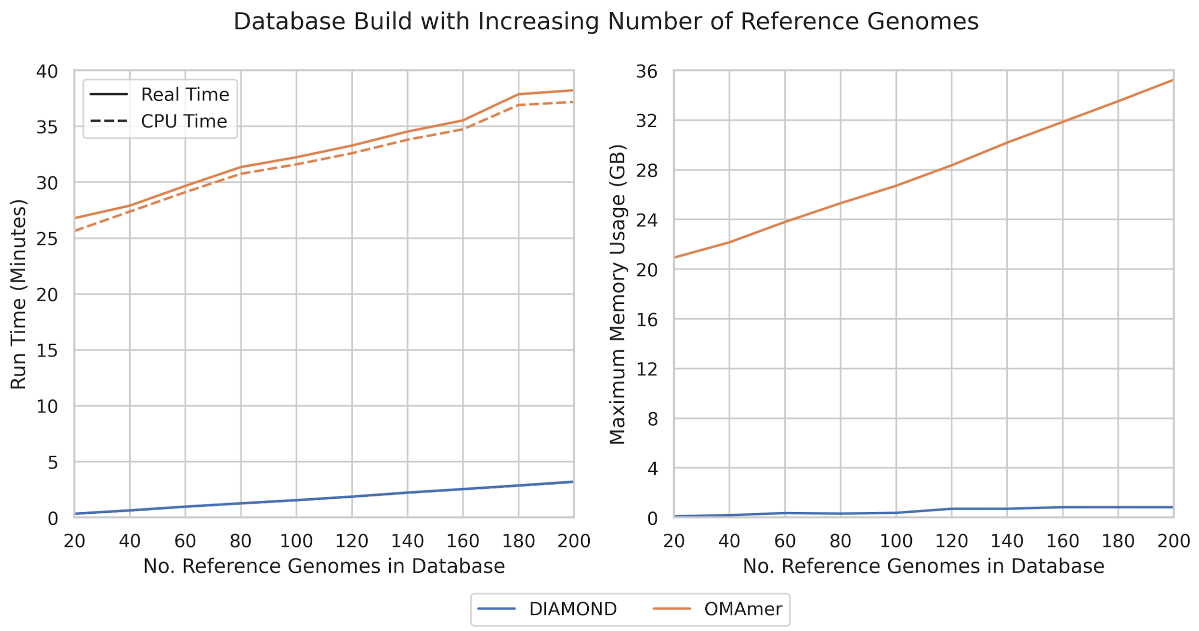

Supp. Figure 6. Run time (left) and maximum memory usage (right) during database build for DIAMOND and OMAmer. Whilst OMAmer is slower and requires more memory due to the increased pre-processing to enable fast lookup time, the increase in time and memory is linear with the number of reference genomes in the resulting database.

Supp. Table 1. Formulae of validation measures

Measure	Formula
Precision	$\frac{\#TPs}{(\#TP\ +\ \#FPs)}$
Recall	$\frac{\#TPs}{(\#TP\ +\ \#FNs)}$
Accuracy	$2x\ \frac{(precision\ *\ recall)}{(precision\ +\ recall)}$

#: number, TPs: true positives, FPs: false positives, FNs: false negatives.

Supp. Table 2. Species used as queries in benchmarks.

**Species**	**Scientific name**	**LCA clade**	**Divergence age (mya)**	**Genome scaffold N50 (kb)**
Spotted Gar	*Lepisosteus oculatus*	*Neopterygii*	320 (Betancur-R *et al.*, 2017)	6928 (Ensembl LepOcu1 assembly)
Platypus	*Ornithorhynchus anatinus*	*Mammalia*	250 (Upham *et al.*, 2019)	992 (Ensembl OANA5 assembly)
Amphioxus	*Branchiostoma floridae*	*Chordata*	600 (Peterson and Eernisse, 2016)	2600 (Putnam *et al.*, 2008)
Gray rockcress	*Arabis alpina*	*Brassicaceae*	27 (Willing *et al.*, 2015)	788 (Willing *et al.*, 2015)
Grape wine	*Vitis vinifera*	*Rosids*	>130 (Jaillon *et al.*, 2007)	2070 (Jaillon *et al.*, 2007)
Amborella trichopoda	*Amborella trichopoda*	*Magnoliopsida*	>160 (Amborella Genome Project, 2013)	4900 (Amborella Genome Project, 2013)

References

Amborella Genome Project (2013) The Amborella genome and the evolution of flowering plants. *Science*, **342**, 1241089.

Betancur-R,R. *et al.* (2017) Phylogenetic classification of bony fishes. *BMC Evol. Biol.*, **17**, 162.

Jaillon,O. *et al.* (2007) The grapevine genome sequence suggests ancestral hexaploidization in major angiosperm phyla. *Nature*, **449**, 463–467.

Peterson,K.J. and Eernisse,D.J. (2016) The phylogeny, evolutionary developmental biology, and paleobiology of the Deuterostomia: 25 years of new techniques, new discoveries, and new ideas. *Org. Divers. Evol.*, **16**, 401–418.

Putnam,N.H. *et al.* (2008) The amphioxus genome and the evolution of the chordate karyotype. *Nature*, **453**, 1064–1071.

Upham,N.S. *et al.* (2019) Inferring the mammal tree: Species-level sets of phylogenies for questions in ecology, evolution, and conservation. *PLoS Biol.*, **17**, e3000494.

Willing,E.-M. *et al.* (2015) Genome expansion of Arabis alpina linked with retrotransposition and reduced symmetric DNA methylation. *Nat Plants*, **1**, 14023.
